# American Association for Emergency Psychiatry Task Force on Medical Clearance of Adult Psychiatric Patients. Part II: Controversies over Medical Assessment, and Consensus Recommendations

**DOI:** 10.5811/westjem.2017.3.32259

**Published:** 2017-05-01

**Authors:** Michael P. Wilson, Kimberly Nordstrom, Eric L. Anderson, Anthony T. Ng, Leslie S. Zun, Jennifer M. Peltzer-Jones, Michael H. Allen

**Affiliations:** * University of Arkansas for Medical Sciences, Department of Emergency Medicine, Little Rock, Arkansas; † University of Colorado School of Medicine, Department of Emergency Medicine, Aurora, Colorado; ‡ Office of Behavioral Health, Department of Human Services, State of Colorado, Denver, Colorado; § University of Maryland, Department of Psychiatry, Cambridge, Maryland; ¶ Uniformed Services School of Medicine, Department of Psychiatry, Bethesda, Maryland; || Chicago Medical School, Department of Emergency Medicine, Chicago, Illinois; # Mount Sinai Hospital, Department of Emergency Medicine, New York, New York; ** Henry Ford Health System, Department of Emergency Medicine, Detroit, Michigan

## Abstract

**Introduction:**

The emergency medical evaluation of psychiatric patients presenting to United States emergency departments (ED), usually termed “medical clearance,” often varies between EDs. A task force of the American Association for Emergency Psychiatry (AAEP), consisting of physicians from emergency medicine, physicians from psychiatry and a psychologist, was convened to form consensus recommendations for the medical evaluation of psychiatric patients presenting to U.S.EDs.

**Methods:**

The task force reviewed existing literature on the topic of medical evaluation of psychiatric patients in the ED and then combined this with expert consensus. Consensus was achieved by group discussion as well as iterative revisions of the written document. The document was reviewed and approved by the AAEP Board of Directors.

**Results:**

Eight recommendations were formulated. These recommendations cover various topics in emergency medical examination of psychiatric patients, including goals of medical screening in the ED, the identification of patients at low risk for co-existing medical disease, key elements in the ED evaluation of psychiatric patients including those with cognitive disorders, specific language replacing the term “medical clearance,” and the need for better science in this area.

**Conclusion:**

The evidence indicates that a thorough history and physical examination, including vital signs and mental status examination, are the minimum necessary elements in the evaluation of psychiatric patients. With respect to laboratory testing, the picture is less clear and much more controversial.

## INTRODUCTION

Emergency physicians (EP) are commonly required to diagnose and treat psychiatric patients.^1^ In 2011, for instance, EPs diagnosed “mental disorders” in approximately 3.9% of patient visits.^2^ Many psychiatric patients presenting to an emergency department (ED) require some form of aftercare (i.e., psychiatric admission, transfer to a psychiatric crisis center, etc.). Thus, EPs are often asked to “medically clear” psychiatric patients.

As EDs often perform assessments of psychiatric patients, who commonly have coexisting medical and psychiatric disease, it is imperative that both emergency and psychiatric physicians find a common language and point of reference to care for these patients. A consequence of not sharing a common treatment algorithm or language is evident in the Tintinalli et al. study, in which 80% of patients listed as “medically clear” on the chart actually had medical disease that should have been identified during a standard history and physical.^3^

EDs are further limited by the capabilities of receiving institutions, as many free-standing psychiatric facilities lack medical equipment and trained staff to care for coexisting medical disease.^4^ EDs have therefore been forced to perform increasingly comprehensive medical screening exams before transferring patients to these units. As funding for psychiatric facilities decreases, the number of psychiatric inpatient beds has declined, which has the deleterious effect of increasing the acuity of psychiatric units both medically and behaviorally. Limited bed availability prolongs lengths of stay (LOS) for psychiatric patients, although it is not known how medical complexity affects availability of psychiatric beds.^5^ As numbers of psychiatric patients in the ED subsequently increase, waiting times and LOS for all ED patients are affected, making this an important issue for all EDs.^6^

This is part II of the American Association for Emergency Psychiatry (AAEP) task force on medical examinations of psychiatric patients presenting to EDs. The task force was composed of EPs, emergency psychiatrists, and an emergency psychologist. Task force members consisted of Michael P. Wilson, Kimberly Nordstrom, Eric L. Anderson, Anthony Ng, Leslie Zun, Jennifer M. Peltzer-Jones, and Michael H. Allen, chosen by the AAEP for their expertise on the topic, all with an extensive background in behavioral emergencies. Consensus was achieved by group discussion and iterative revisions of the written document. The purpose of this task force was to examine the existing evidence, synthesize it into cohesive guidelines, and examine areas for future research in the areas of emergency medicine (EM) and emergency psychiatry. This document was reviewed and approved by the AAEP Board of Directors.

## CONTROVERSIES OVER “MEDICAL CLEARANCE”

There are a number of current areas of controversy in the emergency medical examinations of psychiatric patients:

- defining adequate medical examination for psychiatric patients;
- outlining the role of routine laboratory testing, including urine drug screens and medical algorithms;
- reviewing the standards of the capabilities of psychiatric receiving facilities.

Each of these questions is discussed in turn.

### Defining an Adequate Medical Exam

Several studies have investigated the important elements of emergency medical triage or screening exams for psychiatric patients.^7^–^14^ There is general consensus that psychiatric patients with abnormal vital signs, advanced age (>= 65 years of age), severe agitation, evidence of toxic ingestion, or decreased level of awareness are more likely to have a medical cause for their illness and therefore warrant further testing.^15^ Many authors have also advised formal mental status screenings in the ED, especially for elderly patients, since patients with frank disorientation are more likely to have a medical cause of their symptoms than a psychiatric diagnosis.^16^–^17^ Although there have been few studies investigating differences between screening tools in the ED, one study by Kaufman and Zun found that a six-item questionnaire had a sensitivity of 72% and a specificity of 95% in identifying individuals with severely impaired mental status, took only a few minutes to complete, and was rated as useful by most clinicians administering the test.^8^

Although a prospective randomized trial of the addition of mental status screenings alongside physical exams has never been performed, these studies highlight the importance of a mental status exam in the medical evaluation of psychiatric patients. Expert guidelines, such as those by the American College of Emergency Physicians (ACEP), recommend an assessment of mentation as part of medical screening in EDs.^18^ Although no studies have investigated the use of allied health personnel in the screening of psychiatric patients, most have relied, either explicitly or implicitly, on the judgment of attending EPs or similarly qualified individuals.

### The Role of Routine Laboratory Testing and Medical Algorithms

Whether or not there should be a reasonable suspicion of disease in asymptomatic patients with normal vitals and a psychiatric chief complaint has yielded conflicting results in the EM literature. Nonetheless, at least one study has indicated that many EPs are routinely required to obtain labs for psychiatric patients.^19^ These routine labs generally do not reveal serious disease, especially if the patient is young.^20^–^23^ Olshaker and colleagues, for instance, reported on a series of 65 patients with a coexisting medical condition presenting for a psychiatric complaint.^20^ The authors concluded that history and physical examination alone were able to detect the vast majority of medical illness. Janiak and Atteberry reviewed 502 charts of psychiatric patients who received routine laboratory testing by the psychiatric service and found, with only one exception, no labs ordered routinely would have changed ED management.^21^ Amin and Wang prospectively studied 375 psychiatric patients presenting for medical assessment. In this study, 14.9% of patients had non substance-induced laboratory abnormalities that either occurred in patients with abnormal history or physical exams or were not felt to alter final disposition or contribute to the patient’s presentation.^22^ Korn and colleagues reviewed 212 charts, finding that the initial complaints of these patients correlated directly with the need for additional testing.^23^

A study by Henneman and colleagues, however, reached opposite conclusions.^7^ The authors investigated 100 consecutive patients aged 16–65 who presented to the ED with new-onset psychiatric complaints and no known past psychiatric history. In this cohort, 63 patients were found to have coexisting medical illness. History and physical examination alone suggested disease in only 27 of the 63 patients; the authors concluded that most adult patients with new-onset psychiatric symptoms have a medical etiology and recommended extensive assessment for all patients with new-onset psychiatric complaints.

Unfortunately, the controversy in the literature regarding the importance of physical exams and laboratory testing is difficult to resolve with existing studies such as these, since none of the studies above documented the elements of their physical or mental status examinations. Further, none of these studies investigated whether testing high-risk groups increases the yield of laboratory investigations. Although a definitive answer to the question of testing awaits further research, at least some evidence exists that routine testing adds little to disposition decisions beyond the clinical judgment of an attending EP. Based on evidence of this type, ACEP, in a recent clinical guideline on evaluation of adult psychiatric patients, stated that routine laboratory testing for asymptomatic, alert, cooperative patients was unnecessary.^24^ It is unknown, however, how routine testing may contribute to the identification of chronic coexistent disease such as diabetes or renal failure, which may be more important for provision of care after the ED.

The utility of routine urine drug screens has also been questioned. In theory, provider knowledge of exposure to drugs of abuse could potentially alter diagnosis and disposition to addiction treatment versus a psychiatric setting. This is relevant partly because these settings are funded by different mechanisms in some states. In support of routine testing, studies such as Schuckman et al. have indicated self-reporting of illicit drug use is unreliable in the ED setting.^25^ However, several ED studies have indicated that verification of a patient’s substance use with urine drug screens does not often change ED disposition of psychiatric patients.^26^–^29^ In a prospective study of 392 patients presenting to a psychiatric emergency service, for instance, Schiller and colleagues found 20.8% of patients who denied substance use actually had positive screens, but dispositions did not change between patients in whom a routine urine drug screen was ordered (the mandatory-screen group) and patients in whom it was not (usual-care group).^25^ Similar results were found by Korn and colleagues in a retrospective review of 212 charts, Fortu and colleagues in a retrospective review of 652 charts, and Eisen and colleagues in a prospective study of 133 patients.^23^,^27^–^28^

At least one study has found that when a urine drug screen was checked, it was correct for all five drugs of abuse only in 75.2% of cases, raising questions about the accuracy of the test.^29^ ACEP, in a guideline on evaluation of adult psychiatric patients, stated routine testing for urine drugs of abuse was unnecessary in the ED but offered this only as a Level C recommendation.^18^ Based on these studies, it appears that ED management would not often be changed as a result of urine toxicologic testing. However, if comorbid substance use is detected, it should become a focus of any subsequent treatment. Receiving psychiatric facilities may request this study, as it is time critical and may affect the direction of further mental health treatment. Unfortunately, no studies have examined the cost of performing this test at psychiatric receiving facilities, whether the results of this test would change the subsequent care setting or treatment decisions, or the impact on payment.

Given the often conflicting demands between comprehensive medical testing that is useful to consultants and the desire of many EPs only to obtain testing that will affect their disposition and management in the ED, many authors have advocated the use of medical algorithms that are agreed upon in advance by all parties involved. Zun and colleagues in their work with the Illinois Mental Health Task Force set forth three basic criteria for hospitalization in a state-operated psychiatric facility: evidence of a psychiatric diagnosis severe enough to warrant inpatient hospitalization; clinically-indicated evaluation of any suspected medical illness; and the stability of any medical problems in order to allow both safe transport to the facility and hospitalization at that institution.^10^ Additional guidelines were adopted to specify the term “clinically-indicated evaluation.” In a later study, Zun and Downey performed a retrospective chart review of all ED patients with psychiatric complaints who were transferred to a psychiatric facility after the adoption of the medical clearance protocol, compared to all patients who were transferred before the protocol.^11^–^12^ The total cost of diagnostic testing was $269 per patient after the adoption of the protocol and $352 before, which was a statistically significant difference. The return rate of patients to the ED after the protocol, however, was similar.

Another screening algorithm was recently proposed by Shah and colleagues.^13^ In this study, the authors retrospectively reviewed 485 charts of psychiatric patients who had been evaluated by attending EPs with a five-item screening tool created for psychiatric patients. Patients with a “yes” to all five questions (stable vital signs, prior psychiatric history, alert/oriented × 4, no evidence of acute medical problem, no visual hallucinations) were discharged to a psychiatric receiving facility without further testing. Only six patients (1.2%) with “yes” to all questions required further medical workup and were returned to the ED. No patients required medical or surgical admission.

Despite studies like these, however, a simple medical screening algorithm with broad applicability to psychiatric patients presenting to EDs has yet to be validated or widely adopted.

### The Capabilities of Psychiatric Receiving Facilities

The Emergency Medical Treatment and Labor Act (EMTALA) requires that, for a transfer to be appropriate, the receiving facility must have the capability to treat the patient. For psychiatric facilities, this would imply the capability to treat both medical and psychiatric conditions. However, medical capability varies widely within the range of available psychiatric facilities. The level of capability often affects ED medical screening processes in ways that are not scientific. In 2002, the American Psychiatric Association (APA) task force on psychiatric emergency services set forth clear guidelines for basic capabilities of different types of psychiatric receiving facilities.^30^–^31^ The lowest level of care in this report was termed a psychiatric urgent care facility, which was still required to be able to perform basic medical testing. However, these guidelines have not been widely adopted.

The idea that psychiatric receiving facilities, not attached to a hospital, should meet APA guidelines for operating at the level of a psychiatric urgent care facility or higher has been suggested in the literature,^30^ but did not find consensus in the current work group.

## AAEP CONSENSUS STATEMENT ON MEDICAL EVALUATION OF PSYCHIATRIC PATIENTS

After reviewing existing evidence, the AAEP Task Force makes the following recommendations for the evaluation of psychiatric patients presenting acutely to EDs. In general, there are no randomized clinical trials comparing different strategies for medically assessing psychiatric patients in the ED. Nor are there randomized trials investigating reliable markers of medical illness in the psychiatric patient. Thus, recommendations are based on expert consensus and should be treated as preliminary until further evidence is obtained.

### Recommendation 1

The goal of medical assessment of psychiatric patients in an ED is to identify potential causative factors for a patient’s presenting complaint (i.e., medical mimics) as well as medical problems that will need ongoing care but do not contribute directly to the presenting psychiatric complaint. Examples of the former include encephalopathy, substance intoxication/withdrawal, infections, or central nervous system disease. Examples of the latter include chronic obstructive pulmonary disease or diabetes. EDs should perform an appropriate medical screening exam and appropriate documentation for the presenting complaint. If there is a question whether the patient has delirium or a psychiatric disorder, this patient should be medically observed or hospitalized.

### Recommendation 2

Further medical evaluation should be considered for patients who have (1) new-onset psychiatric symptoms after the age of 45 years,^32^–^33^ (2) advanced age (65 years of age and older),^34^–^35^ (3) cognitive deficits or delirium, (4) positive review of systems indicative of a physical etiology, such as cough and fever, (5) focal neurological findings or evidence of head injury, (6) substance intoxication, withdrawal, or exposure to toxins/drugs, (7) decreased level of awareness, or (8) other indications, such as abnormal vital signs that direct further assessment. An example includes a urinalysis in elderly patients with dysuria (or other sign/symptom of urinary tract infection) and new-onset altered mental status. As an aside, obtaining a urinalysis for all elderly patients with altered mental status but no symptoms specific to urinary tract infection may lead to premature treatment as asymptomatic pyuria is common in elderly patients.^37^ The cause of the mental status change may lie elsewhere and require further workup.

### Recommendation 3

The term “medical clearance” should not be used as it minimizes the presence of chronic medical problems and is not in line with current ED terminology. Instead, all patients seen in medical ED prior to transfer to psychiatric emergency services, psychiatric inpatient units, or other psychiatric settings must be evaluated medically. In place of a statement that the patient is “medically clear,” a transfer note should accompany the patient indicating the patient is medically stable and appropriate for treatment in a psychiatric setting, i.e., that their behavioral disturbance is unlikely to be due to a medical condition or physical trauma, and that medical/surgical treatment for any concomitant conditions is within the capabilities of the receiving facility.

This last statement implies that the continuing medical care required has been defined by the sending facility and that the necessary care will be available in a timely fashion at the receiving facility. The transfer note should include the details of the assessment performed, the results, and the medical decision-making that occurred to deem the patient appropriate for transfer with recommendations for the further assessment and care of any active medical problems. It may be necessary to document that the patient is medically stable for transfer per EMTALA guidelines, though these guidelines ought to be considered the minimum rather than the standard level of evaluation.

### Recommendation 4

Universal screening of the psychiatric patient must, at minimum, include vitals, history, a physical examination, and assessment of mentation. A brief cognitive exam is preferred over a simple assessment of mentation, as the latter typically includes only a statement regarding the patient’s level of alertness and orientation. Ideally, this cognitive exam should include assessment of attention, executive function, orientation, and recent memory. This detailed evaluation of cognitive status may be performed by clinicians, such as mental health consultants or allied health staff, who have been trained in mental health testing. The decision for further evaluation, however, should be based on the EP’s assessment.

### Recommendation 5

Since many psychiatric settings have limited medical capability, e.g., phlebotomy available only at certain times on weekdays, accepting physicians may ask that “routine” tests be done before the accepted patient arrives at the facility. These requests should be honored where possible, but should not delay the transfer of patients who are otherwise deemed medically appropriate for transfer. Clinically directed ED laboratory testing should be reviewed prior to transfer. Routine laboratory testing may be reported after the patient is transferred as long as there is a communication process with the accepting facility.

### Recommendation 6

EDs should work cooperatively with their psychiatric receiving facilities to develop protocols that identify low- and high-risk categories or conditions, and the procedures required for each category at each facility. Testing such as laboratory evaluations or neuroimaging may be deferred for some categories and required in others as in recommendation 2.

### Recommendation 7

In resolving disputes over whether a patient’s condition is appropriate for psychiatric transfer and treatment, clinicians at both the accepting and receiving facilities should carefully review the specific patient’s vital signs, history, and physical exam. In this clinical encounter, it is important to be clinically reasonable about the odds of suspected non-psychiatric diagnoses. It is neither efficient nor effective for psychiatric staff to require that statistically unlikely diagnoses be “ruled out,” e.g., systemic lupus erythematosus in a 20-year-old male with low energy and a rash. On the other hand, ED staff should consider non-psychiatric diagnoses that mimic psychiatric conditions, such as hypothyroidism causing depressive symptoms. The treatment is different than for a primary depression (such as major depressive disorder).

### Recommendation 8

There is a great need for additional research in the area of medical screening. We recommend the following:

1. What are the essential elements of a history that might efficiently form the basis for universal screening of psychiatric patients? What are the vital elements of the physical exam?
2. What are the criteria that define groups at high risk for medical disease? Are there criteria that should be considered indications for more extensive evaluation in an ED? Are there critical values in vital signs or laboratory examinations that predict difficulty in managing the patient after leaving the ED?
3. What is role of urine toxicology and would point-of-care testing significantly alter the time required and the related cost benefit analysis?
4. Does the regionalization or specialization of emergency psychiatric receiving facilities, similar to regional trauma centers, provide better care for mental health patients? Could direct assessment by receiving facilities via telemedicine improve the processes and obviate the need for some procedures and transfers?
5. What is the most effective system for medical screening? In particular, qualitative studies are needed of receiving hospitals, as well as the match between the sending ED’s assessment, the transfer plan, and the receiving service’s assessment and capabilities of managing the patient.

## CONCLUSION

The testing of psychiatric patients who present to the ED is an area of controversy, in part because there is little evidence to inform most elements of the evaluation process. After reviewing existing evidence, the task force believes there may be patients who can safely be considered low risk either for medical mimics of psychiatric disease or for co-existing medical disease. These patients generally have each of the following characteristics: young, present to the ED with an isolated psychiatric complaint, have a past history of psychiatric disease, are not using illicit substances, have normal vitals, and have a history and physical exam that does not suggest medical illness. Conversely, there likely exists a group of patients at higher risk both for medical mimics of psychiatric disease and for co-existing medical illness. These patients may have any of the following: older age, abnormal vitals and/or disorientation, no previous history of psychiatric disease, or a history and/or physical exam that suggests medical illness. In these patients, thorough medical assessment is likely indicated. The exact criteria defining these two groups are not well specified but should be subjected to further research. The essential elements of assessment of all psychiatric patients, regardless of risk of co-existing medical illness, are also not generally agreed upon. The task force believes further research in this area is necessary. In the interim, EDs should work cooperatively with their psychiatric facilities to develop protocols that allow both adequate medical screening of psychiatric patients and their efficient disposition from the ED.
